# Case Report: Unmasking catatonia in adolescents: a potential triggering role of corticosteroids in two clinical cases

**DOI:** 10.3389/fpsyt.2026.1945779

**Published:** 2026-09-03

**Authors:** Margherita De Biase, Roberto Averna, Gianluca Sesso, Milena Labonia, Stefano Vicari

**Affiliations:** 1 Child and Adolescent Neuropsychiatry Unit, Department of Neuroscience, IRCCS Children Hospital Bambino Gesù, Rome, Italy; 2 Department of Life Sciences and Public Health, Catholic University of the Sacred Heart, Rome, Italy

**Keywords:** adolescents, catatonia, clozapine, corticosteroids, neuroinflammation

## Abstract

**Background:**

Catatonia is a transdiagnostic syndrome marked by motor, behavioral, and autonomic disturbances. Corticosteroids, commonly used to treat suspected neuroinflammatory conditions, may rarely precipitate or exacerbate psychiatric symptoms, including catatonia, in children and adolescents.

**Methods:**

We describe two adolescent girls who developed catatonia following corticosteroid therapy for suspected encephalitis, despite negative cerebrospinal fluid and blood markers. Clinical courses, diagnostic workups, and therapeutic strategies (including benzodiazepines, antipsychotics, and immunomodulatory treatments) are detailed. A literature review of steroid-induced neuropsychiatric complications in pediatric populations was also performed.

**Results:**

Both patients experienced temporal association between corticosteroid exposure and onset of catatonia. Standard first-line treatments were insufficient, and stabilization required antipsychotic therapy, including clozapine. Gradual improvement in baseline clinical status and recovery of lost functional abilities were observed during follow-up. These cases underscore that corticosteroid-induced catatonia, though rare compared to steroid-related psychosis, can occur in adolescents and may complicate the differential diagnosis of neuropsychiatric syndromes.

**Conclusions:**

While corticosteroids remain essential for treating neuroinflammatory conditions, clinicians should be aware of the potential for severe psychiatric adverse effects, including catatonia, in pediatric patients. Careful differential diagnosis, interdisciplinary collaboration, and timely psychiatric intervention are critical to avoid iatrogenic worsening. Further research is needed to clarify underlying neurobiological mechanisms and identify markers of vulnerability, promoting safer and more personalized treatment approaches in children and adolescents.

## Introduction

Catatonia is a complex syndrome characterized by a range of motor, behavioral, and autonomic abnormalities. Mutism, negativism, posturing and stupor are signs that most often identify the syndrome (1). Although traditionally associated with schizophrenia, DSM-5 reports that catatonia may occur in the context of a wide spectrum of psychiatric, neurological, and medical conditions, highlighting its transdiagnostic nature (2).

In adult populations with major psychiatric disorders, including schizophrenia, affective disorders, and other psychotic illnesses, studies have reported catatonia incidence rates ranging from 10% to 37.7% (3–5), with a stronger association observed between catatonia and mood disorders compared to other diagnoses. Research on the incidence and clinical features of catatonia in pediatric populations remains scarce. The study by Cohen et al. (6) reported a catatonia incidence of 0.6% in inpatient adolescents. In contrast, Thakur (7) analyzed 198 pediatric psychiatric outpatients and found schizophrenia as the most frequent diagnosis associated with catatonia, unlike in adult populations. Furthermore, the incidence of catatonia reported by Thakur was higher than that found by Cohen et al., with 5.5% of the entire sample and 17.7% of patients diagnosed with affective or non-affective psychotic disorders presenting with catatonia.

In the pediatric population, catatonia is observed in neurodevelopmental disorders (notably autism) (8), early-onset schizophrenia and other psychoses (6, 9), mood disorders (10, 11), and occasionally in anxiety, dissociative, or eating disorders. It can also arise from general medical conditions such as epilepsy, encephalitis (12), metabolic (13, 14) and genetic diseases, or after exposure to substances including MDMA (15), PCP (16), and certain prescribed drugs. Neuroleptics may induce catatonia or neuroleptic malignant syndrome (17). Among pharmacological agents, corticosteroids represent a rare but clinically relevant trigger. Notably, in the diagnostic evaluation of psychiatric symptomatology suspected to have a neurological etiology, such as autoimmune or infectious encephalitis, corticosteroids are frequently administered to suppress neuroinflammation. However, when the underlying condition is of primary psychiatric origin, corticosteroid exposure may paradoxically exacerbate symptoms and precipitate catatonia.

The exact mechanism by which corticosteroids may induce catatonia has not yet been fully understood. Current evidence suggests that corticosteroid exposure may act through neurobiological pathways implicated in psychiatric disorders in which catatonia is observed. Specifically, corticosteroids can mimic stress and interfere with the regulation of the hypothalamic–pituitary–adrenal (HPA) axis, thereby increasing vulnerability to psychosis in accordance with the stress–vulnerability model of schizophrenia (18, 19). Prolonged cortisol release disrupts HPA axis feedback mechanisms, leading to dysregulation and sustained hyperactivation (20). This process results in persistently elevated cortisol levels and excessive stimulation of glucocorticoid receptors (GRs), particularly within the hippocampus (21, 22). Glucocorticoids have been associated with dose-dependent hippocampal damage, although this remains debated and may require concomitant stress exposure (23). Importantly, GR overactivation impairs neurogenesis and contributes to hippocampal atrophy, mechanisms that have been consistently linked to the development of major depressive disorder (24–26).

In this report, we describe two adolescent patients who developed catatonia following corticosteroid treatment initiated for suspected encephalitis. We outline their clinical course, diagnostic work-up, and therapeutic management, and discuss the implications for clinical practice and early recognition of corticosteroid-related neuropsychiatric complications in the pediatric population.

## Case series

### Patient A

A 13-year-old girl with no prior psychiatric history presented with subacute behavioral and mood changes following an episode of febrile otitis media. The temporal association between the infectious episode and the subsequent neuropsychiatric deterioration, together with the rapid progression of symptoms, initially raised concern for an underlying inflammatory or autoimmune encephalitic process. Symptoms included disinhibition, distractibility, affective lability, persecutory ideation, and bizarre behavior, progressively evolving over the course of one month and culminating in frank psychotic features. Due to initial clinical suspicion of infectious or autoimmune encephalitis, she was admitted to a neurology unit and underwent extensive diagnostic workup, which was largely unremarkable. Her family history was notable for autoimmune conditions (including systemic lupus erythematosus, vasculitis, and Hashimoto’s thyroiditis) as well as a depressive disorder in a paternal cousin. Brain MRI, EEG, chest X-ray, and abdominal ultrasound, performed also to exclude ovarian teratoma as a potential trigger of anti-NMDAR encephalitis, were unremarkable. Cerebrospinal fluid analysis was negative for infectious and autoimmune markers, except for weak positivity for anti-GD1a, anti-GT1b, and anti-GM2 antibodies.

Empirical immunotherapy was initiated with high-dose intravenous methylprednisolone (1 g/day for 3 days; Days 0–2), followed by a 12-day tapering regimen (Days 3–14). The planned corticosteroid regimen was completed on Day 14. Although a mild initial improvement was observed during corticosteroid administration, a marked clinical deterioration developed shortly after corticosteroid discontinuation, with progression of neuropsychiatric symptoms and emergence of a severe catatonic state. At admission to the neurology unit on Day 15, she was markedly unresponsive and unable to move spontaneously, with mutism, postural rigidity, food refusal, impaired oral medication intake, loss of sphincter control, and marked withdrawal.

During the following 15 days (Days 15–29), treatment with diazepam and IVIG was administered without clinically meaningful benefit. On Day 30, because of persistent and worsening neurological symptoms, the patient was transferred to our Neurology Department for further diagnostic and therapeutic management. Given the severe catatonic state, high-dose oral lorazepam (15 mg/day) was initiated on Day 30. A second diagnostic workup, including brain MRI, EEG, and lumbar puncture, was performed. From Day 30 to Day 44, in view of the persistent clinical picture and the possibility of seronegative autoimmune encephalitis, a further course of immunomodulatory treatment was administered over approximately 15 days, including IVIG, corticosteroids, and plasmapheresis. No antipsychotic medication was administered during this phase because of the ongoing concern for an underlying neurological disorder. A gradual but modest clinical improvement was initially observed, followed by a further deterioration. Around Day 45, the patient appeared markedly perplexed, with minimal spontaneous speech. Neurologically, she showed psychomotor slowing, paramimic facial expressions, pseudo-choreic movements, and a tendency to briefly maintain abnormal postures, although waxy flexibility was absent. Passive behavior was prominent, with occasional echopraxia and echolalia. Formal assessment of thought content and perceptual disturbances was not possible because of the severity of the clinical condition. Nevertheless, a pervasive paranoid atmosphere was suspected, accompanied by avoidance and social withdrawal. At this stage, the Bush–Francis Catatonia Rating Scale (BFCRS) (27) was assessable with a total score of 26, indicating the persistence of clinically significant catatonic symptoms.

On Day 46, the patient was transferred to our Child and Adolescent Neuropsychiatry Unit. She remained hospitalized for 15 days (Days 46–60). A comprehensive psychopathological assessment supported a diagnosis of unspecified psychotic disorder. During hospitalization, the patient exhibited recurrent crying episodes and periods of intense fear and distress. As her clinical condition gradually allowed greater verbal interaction, she progressively disclosed persecutory and delusional thought content, which became increasingly evident during the course of the hospitalization.

On Day 46, intramuscular aripiprazole and intravenous diazepam were initiated because she refused oral medication. On Day 52, after 6 days of treatment and once oral medication became feasible, therapy was switched to oral risperidone and lorazepam. Partial improvement was observed over the following 6 days, however, clinically significant psychotic and catatonic symptoms persisted. On Day 58, risperidone was therefore discontinued and replaced with clozapine, which was progressively titrated to 150 mg/day. Lithium was initiated on Day 60 as an adjunctive mood-stabilizing treatment, particularly in view of the affective and behavioral dysregulation observed throughout the clinical course.

The patient was discharged on Day 60, with the aim of monitoring whether return to her familiar home environment would facilitate further recovery.

At the 17-day post-discharge follow-up, the patient was more responsive and less rigid. Speech was present but remained limited, with delayed responses and occasional incongruent answers. At this stage, the Bush–Francis Catatonia Rating Scale (BFCRS) was assessable with a total score of 9, indicating the improvement of catatonic symptoms.

She continued to experience marked emotional distress and persecutory and guilt-related delusions, including beliefs that she had committed a grave sin and had killed and cannibalized her parents, who had subsequently resurrected and been replaced (Capgras delusion). She also expressed fear of being eaten herself and showed possible visual or auditory misperceptions, with suspicious scanning of the environment. Emotional lability and frequent crying persisted.

In response, clozapine was increased to 200 mg/day, lithium to 166 mg/day, and lorazepam to 6 mg/day. Five days after these adjustments, a marked reduction in delusional symptoms and significant overall clinical improvement were observed. Given the concomitant adjustment of multiple medications, this improvement cannot be attributed to any single pharmacological intervention. Follow-up was subsequently continued through community mental health services.

Notably, the onset of catatonia followed corticosteroid treatment, raising questions about the potential role of corticosteroids in the clinical course.

### Patient B

The second patient was a 15-year-old girl with no relevant early developmental problems and a previously described personality marked by social engagement and perfectionism.

Following an emotionally distressing interpersonal experience, she progressively developed social withdrawal and behavioral changes, including persistent preoccupation with a public figure and excessive use of social media.

Approximately two months later, incongruent affect, auditory hallucinations, behavioral dysregulation, and agitation emerged. During the following months, behavioral dysregulation, irritability, episodes of anger, and agitation progressively increased.

Approximately eight months after symptom onset, she underwent her first child and adolescent psychiatric assessment. At the assessment, symptoms of emotional and behavioral dysregulation, obsessive-compulsive symptomatology, and psychotic symptoms (including auditory hallucinations and behavioral disorganization) were observed. Treatment with risperidone, paroxetine, and diazepam was therefore initiated. Paroxetine was subsequently discontinued because of suspected clinical worsening, and a trial of sertraline was also stopped because of increased agitation. Clinical stabilization was reported approximately ten months after symptom onset while receiving risperidone 1–1.75 mg/day.

She showed temporary stabilization but three months later she was admitted to a child and adolescent neuropsychiatry unit for diagnostic reassessment and for evaluation of a recrudescence of psychotic symptoms. Brain MRI revealed a modest anterior hippocampal volume reduction of unclear pathological significance; EEG and bloodwork were unremarkable. Cognitive assessment suggested borderline intellectual functioning, although interpretation was limited by active psychotic symptoms. A diagnosis of schizophrenia was confirmed.

Despite this diagnosis, the atypical and severe clinical course and lack of response to antipsychotic treatment prompted consideration of a possible neuroinflammatory contribution. In this context, one month after admission, corticosteroid cycles were prescribed during the subsequent two months, administered at approximately 21-day intervals. During this period, a clinical worsening was reported, characterized by increased agitation, loss of sphincter control, and the emergence of catatonic features, including mutism, stereotyped behaviors, reduced facial expressivity, and psychomotor slowing, which progressively worsened in temporal association with steroid treatment. At this stage, the Bush–Francis Catatonia Rating Scale (BFCRS) was assessable, with a total score of 16.

Following this deterioration, corticosteroid treatment was discontinued. Risperidone, which had been maintained at approximately 2 mg/day, was subsequently replaced with clozapine because of insufficient clinical response, with clozapine titrated to 125 mg/day. During the subsequent follow-up, substantial functional impairment persisted. She had not attended school for approximately two years, had no significant social activities, and had lost previously acquired sphincter control. At clinical assessment, she was poorly cooperative, with limited spontaneous speech, reduced facial expressivity, stereotyped trunk movements, impaired self-care, and an irritable and depressed mood. Her parents continued to report auditory hallucinations, while thought content remained largely inaccessible.

Overall, the clinical course was characterized by a progressive psychotic disorder followed by marked clinical exacerbation and emergence of catatonic features during the period temporally associated with corticosteroid exposure. Although a causal relationship cannot be established, the temporal association raised the possibility of corticosteroid-related worsening of the neuropsychiatric syndrome.

During the post-discharge follow-up, a gradual improvement in baseline clinical conditions and a progressive recovery of the autonomies lost during the acute phase of the disorder were observed.

## Discussion

The present case series illustrates the potential of corticosteroids to precipitate or exacerbate catatonia in adolescents with emerging psychiatric disorders. Although catatonia is increasingly recognized as a transdiagnostic syndrome, its occurrence in temporal association with corticosteroid administration has been only rarely reported, particularly in pediatric populations. Both patients in our report developed catatonia following corticosteroid exposure in the context of suspected encephalitis, with subsequent clinical courses more consistent with primary psychiatric disorders. This highlights the importance of considering corticosteroid-related neuropsychiatric effects in the differential diagnosis, especially when the neurological work-up is inconclusive.

The literature on steroid-induced neuropsychiatric complications indicates that corticosteroids can elicit a wide range of affective, behavioral, and cognitive symptoms, including mania, depression, and psychosis, and, more rarely, catatonia. Serious psychiatric adverse effects have been estimated to occur in up to 6% of exposed patients, with dose being the strongest predictor of risk. The incidence of psychiatric symptoms has been reported to increase from 1.3% with prednisone doses of ≤40 mg/day, to 4.6% with doses of 41–80 mg/day, and to 18.4% with doses of ≥80 mg/day. Nevertheless, neuropsychiatric effects have also been reported at substantially lower doses, with symptoms described at doses as low as 2.5 mg/day of prednisolone, indicating that no clear dose threshold can reliably exclude the occurrence of psychiatric adverse effects (28). In adults, the clinical picture is dominated by psychotic and mood-spectrum manifestations, with catatonia representing only isolated reports.

By contrast, pediatric evidence remains extremely limited but clinically informative. Hall et al. (1979) (29) already noted female sex, psychiatric history, and high-dose prednisone as relevant risk factors, and described a 17-year-old girl who developed steroid psychosis while receiving cortisone for asthma. Sullivan and Dickerman (1979) (30) provided the only clear pediatric report of catatonia, describing an 11-year-old with systemic lupus erythematosus treated with prednisone who developed paranoid delusions, mutism, and catatonia at the fourth month of therapy; symptoms resolved after dose tapering. Other pediatric cases have almost invariably concerned psychosis: Ducore (1983) (31) reported acute psychotic episodes in two adolescents with acute lymphoblastic leukemia under induction therapy; Dawson (1998) (32) and French (2003) (33) each described psychotic symptoms in 8-year-olds treated with corticosteroids for asthma; Turktas (1997) (34) reported hallucinations and affective lability in a 14-year-old receiving beclomethasone; Bag (2012) (35) highlighted a 12-year-old boy with steroid-induced psychosis requiring risperidone; and Ularntinon (2010) (36) documented mood and psychotic disturbances in three children with leukemia, successfully managed with risperidone. Additional adolescent cases, such as those by Fleming (2005) (37) and Demir (2012) (38), further reinforce the predominance of psychosis over catatonia in the pediatric population.

These reports suggest that while psychotic symptoms constitute the most frequent severe psychiatric adverse reaction to corticosteroids in children and adolescents, catatonia, so far convincingly described only by Sullivan, remains an exceptional but possible presentation. Our cases contribute to this very limited body of evidence by documenting corticosteroid-associated catatonia in adolescence, a developmental stage characterized by heightened vulnerability due to ongoing neurobiological maturation.

To further assess the likelihood of a causal relationship between corticosteroid exposure and the emergence or exacerbation of catatonia, the Naranjo Adverse Drug Reaction Probability Scale was retrospectively applied to both patients. Patient A obtained a score of 5, corresponding to a probable adverse drug reaction, whereas Patient B obtained a score of 3, corresponding to a possible adverse drug reaction. These findings support a clinically relevant temporal association between corticosteroid exposure and the subsequent neuropsychiatric deterioration, while also highlighting the different strength of the causal inference in the two cases. The lower score in Patient B reflects the presence of important alternative explanations, including the underlying psychotic disorder and the incomplete availability of detailed information regarding corticosteroid exposure. The Naranjo assessment therefore supports, but does not establish, a causal relationship between corticosteroid treatment and catatonia.

The mechanisms likely involve corticosteroid-induced dysregulation of the hypothalamic–pituitary–adrenal (HPA) axis, sustained hypercortisolemia, and glucocorticoid receptor overactivation, leading to downstream effects on hippocampal structure and function, neurogenesis, and neurotransmission. These biological pathways may interact with pre-existing vulnerabilities, such as subtle neurocognitive alterations, family history of autoimmunity, or affective susceptibility, to lower the threshold for catatonia.

From a therapeutic perspective, the pediatric cases reported in the literature underline both the variability of steroid-related psychiatric presentations and the challenges of their management. In most children and adolescents, psychotic symptoms tended to resolve after dose reduction or discontinuation of corticosteroids, sometimes in combination with first-generation antipsychotics such as chlorpromazine (Hall, Ducore, Dawson, French) or, more recently, with atypical agents including risperidone or quetiapine (Ularntinon, Bag, Demir). In contrast, catatonia, so far convincingly described only in the case of Sullivan and Dickerman, improved with steroid tapering alone, without the need for antipsychotics.

Our cases differ in that lorazepam, the first-line treatment for catatonia, was insufficient, and immunomodulatory interventions were also ineffective. Antipsychotics, including clozapine, were required to achieve a progressive stabilization. This discrepancy with prior pediatric reports may reflect heterogeneity in underlying vulnerability, illness context, and exposure characteristics.

Taken together, these findings highlight the clinical dilemma faced when corticosteroids are administered empirically for suspected neuroinflammatory conditions. In ambiguous presentations, corticosteroid therapy may prove deleterious, especially if the underlying etiology is psychiatric. Awareness of the potential for corticosteroid-induced catatonia, even if rare compared to psychosis, is therefore crucial in the pediatric setting. Our cases reinforce the importance of interdisciplinary collaboration, cautious interpretation of diagnostic results, and careful consideration of the risks and benefits of corticosteroid use in children and adolescents presenting with acute psychiatric syndromes. Nonetheless, this should be balanced with the awareness of the importance of early treatment with corticosteroids in cases of serum- and CSF-negative encephalitis.

## Conclusions

Although corticosteroids are widely used and often essential in the management of neuroinflammatory conditions, they may rarely precipitate severe neuropsychiatric complications, including catatonia. Reports of steroid-induced catatonia are extremely limited, particularly in children and adolescents, with only isolated pediatric cases described to date. Our two cases therefore expand on the scarce literature and highlight that catatonia should be considered as a potential adverse effect of corticosteroid therapy in the pediatric population.

These observations underscore the need for heightened clinical awareness when prescribing corticosteroids in patients with unclear neuropsychiatric presentations. Careful differential diagnosis and close collaboration between neurology and psychiatry are crucial to avoid iatrogenic worsening of psychiatric illness. When catatonia emerges in temporal association with corticosteroid use, early recognition and appropriate psychiatric intervention are essential to improving outcomes.

Further research is required to elucidate the neurobiological mechanisms underlying steroid-induced catatonia and to identify clinical or biological markers of vulnerability in pediatric patients. Increased awareness and systematic reporting of such cases may ultimately help guide safer and more personalized treatment approaches.

## Data Availability

The raw data supporting the conclusions of this article will be made available by the authors, without undue reservation.

## References

[1] Rogers JP Zandi MS David AS . The diagnosis and treatment of catatonia. Clin Med (Lond). (2023) 23:242–5. doi: 10.7861/clinmed.2023-0113 37236789 PMC11046566

[2] Tandon R Heckers S Bustillo J Barch DM Gaebel W Gur RE . Catatonia in DSM-5. Schizophr Res. (2013) 150:26–30. doi: 10.1016/j.schres.2013.04.034 23806583

[3] Banerjee A Sharma LN . Catatonia incidence in acute psychiatric admissions. Indian J Psychiatry. (1995) 37:35–40. 21743713 PMC2970947

[4] Rosebush PI Hildebrand AM Furlong BG Mazurek MF . Catatonic syndrome in a general psychiatric population: frequency, clinical presentation, and response to lorazepam. J Clin Psychiatry. (1990) 51:357–62. 2211547

[5] Bräunig P Kruger S Shugar G . Prevalence and clinical significance of catatonic symptoms in mania. Compr Psychiatry. (1998) 39:35–46. doi: 10.1016/s0010-440x(98)90030-x 9472454

[6] Cohen D Flament M Dubos P Basquin M . Catatonic syndrome in young people. J Am Acad Child Adolesc Psychiatry. (1999) 38:1040–6. doi: 10.1097/00004583-199908000-00021 10434497

[7] Thakur A Jagadheesan K Dutta S Sinha VK . Incidence of catatonia in children and adolescents in a paediatric psychiatric clinic. Aust N Z J Psychiatry. (2003) 37:200–3. doi: 10.1046/j.1440-1614.2003.01125.x 12656960

[8] Wing L Shah A . Catatonia in autistic spectrum disorders. Br J Psychiatry. (2000) 176:357–62. doi: 10.1192/bjp.176.4.357 10827884

[9] Green WH Padron-Gayol M Hardesty AS Bassiri M . Schizophrenia with childhood onset: a phenomenological study of 38 cases. J Am Acad Child Adolesc Psychiatry. (1992) 31:968–76. doi: 10.1097/00004583-199209000-00027 1400132

[10] Kinrys PF Logan KM . Periodic catatonia in adolescent. J Am Acad Child Adolesc Psychiatry. (2001) 40:741–2. doi: 10.1097/00004583-200107000-00007 11437011

[11] Powell JC Silveira WR Lindsay R . Pre-pubertal depressive stupor: a case report. Br J Psychiatry. (1988) 153:689–92. doi: 10.1192/bjp.153.5.689 3255459

[12] Ainsworth P . A case of lethal catatonia in a 14-year-old girl. Br J Psychiatry. (1987) 150:110–2. doi: 10.1192/bjp.150.1.110 3651657

[13] Davis EJB Borde M . Wilson's disease and catatonia. Br J Psychiatry. (1993) 162:256–9. doi: 10.1192/bjp.162.2.256 8435699

[14] Rosebush PI McQueen GM Clarke JT Callahan JW Starsberg PM Mazurek MF . Late-onset Tay-Sachs disease presenting as catatonic schizophrenia: diagnostic and treatment issues. J Clin Psychiatry. (1995) 56:347–53. 7635850

[15] Maxwell DL Polkey MI Henry JA . Hyponatraemia and catatonic stupor after taking 'ecstasy'. BMJ. (1993) 307:1399. doi: 10.1136/bmj.307.6916.1399 7903884 PMC1679614

[16] Baldrige EB Bessen HA . Phencyclidine. Emerg Med Clin North Am. (1990) 8:541–50. 2201519

[17] Johnson GC Manning DE . Neuroleptic-induced catatonia: case report. J Clin Psychiatry. (1983) 44:310–2. doi: 10.1016/0278-2316(89)90017-0 6874654

[18] Walker E Kestler L Bollini A Hochman KM . Schizophrenia: etiology and course. Annu Rev Psychol. (2004) 55:401–30. doi: 10.1146/annurev.psych.55.090902.141950 14744221

[19] Needleman H Evans M Roberts H Sweney M Bodensteiner JB . Effects of postnatal dexamethasone exposure on the developmental outcome of premature infants. J Child Neurol. (2008) 23:421–4. doi: 10.1177/0883073807309232 18079310

[20] Ding W Wang L Li L Li H Wu J Zhang J . Pathogenesis of depression and the potential for traditional Chinese medicine treatment. Front Pharmacol. (2024) 15:1407869. doi: 10.3389/fphar.2024.1407869 38983910 PMC11231087

[21] Kamran M Bibi F Ur Rehman A Morris DW . Major depressive disorder: existing hypotheses about pathophysiological mechanisms and new genetic findings. Genes (Basel). (2022) 13:646. doi: 10.3390/genes13040646 35456452 PMC9025468

[22] Wray NR Ripke S Mattheisen M Trzaskowski M Byrne EM Abdellaoui A . Genome-wide association analyses identify 44 risk variants and refine the genetic architecture of major depression. Nat Genet. (2018) 50:668–81. doi: 10.1038/s41588-018-0090-3 29700475 PMC5934326

[23] Maggio N Segal M . Striking variations in corticosteroid modulation of long-term potentiation along the septotemporal axis of the hippocampus. J Neurosci. (2007) 27:5757–65. doi: 10.1523/jneurosci.0155-07.2007 17522319 PMC6672761

[24] Anacker C Zunszain PA Carvalho LA Pariante CM . The glucocorticoid receptor: pivot of depression and of antidepressant treatment? Psychoneuroendocrinology. (2011) 36:415–25. doi: 10.1016/j.psyneuen.2010.03.007 20399565 PMC3513407

[25] Varghese FP Brown ES . The hypothalamic-pituitary-adrenal axis in major depressive disorder: a brief primer for primary care physicians. Prim Care Companion J Clin Psychiatry. (2001) 3:151–5. doi: 10.4088/pcc.v03n0401 15014598 PMC181180

[26] Ciriaco M Ventrice P Russo G Scicchitano F Mazzitello C Scicchitano C . Corticosteroid-related central nervous system side effects. J Pharmacol Pharmacother. (2013) 4:S94–8. doi: 10.4103/0976-500X.120975 24347992 PMC3853679

[27] Bush G Fink M Petrides G Dowling F Francis A . Catatonia. I. Rating scale and standardized examination. Acta Psychiatr Scand. (1996) 93:129–36. doi: 10.1111/j.1600-0447.1996.tb09814.x 8686483

[28] Dubovsky AN Arvikar S Stern TA Axelrod L . The neuropsychiatric complications of glucocorticoid use: steroid psychosis revisited. Psychosomatics. (2012) 53:103–15. doi: 10.1016/j.psym.2011.12.007 22424158

[29] Hall RC Popkin MK Stickney SK Gardner ER . Presentation of the steroid psychoses. J Nerv Ment Dis. (1979) 167:229–36. doi: 10.1097/00005053-197904000-00006 438794

[30] Sullivan BJ Dickerman JD . Steroid-associated catatonia: report of a case. Pediatrics. (1979) 63:677–9. doi: 10.1542/peds.63.4.677 440886

[31] Ducore JM Waller DA Emslie G Bertolone SJ . Acute psychosis complicating induction therapy for acute lymphoblastic leukemia. J Pediatr. (1983) 103:477–80. doi: 10.1016/S0022-3476(83)80432-6 6577167

[32] Dawson KL Carter ER . A steroid-induced acute psychosis in a child with asthma. Pediatr Pulmonol. (1998) 26:362–4. doi: 10.1002/(sici)1099-0496(199811)26:5<362::aid-ppul10>3.0.co;2-g 9859907

[33] French J Khan A White H . Steroid induced psychosis in an asthmatic child: case report and 10 year literature review. Can Child Adolesc Psychiatr Rev. (2003) 12:117–8. doi: 10.1558/sll.2004.11.1.131 PMC2533831 19030155

[34] Türktas L Gücüyener K Ozden A . Medication-induced psychotic reaction. J Am Acad Child Adolesc Psychiatry. (1997) 36:1017–8. doi: 10.1097/00004583-199708000-00006 9256579

[35] Bag O Erdoğan I Onder ZS Altinoz S Ozturk A . Steroid-induced psychosis in a child: treatment with risperidone. Gen Hosp Psychiatry. (2012) 34(1):103.e5–6. doi: 10.1016/j.genhosppsych.2011.09.003 22014832

[36] Ularntinon S Tzuang D Dahl G Shaw RJ . Concurrent treatment of steroid-related mood and psychotic symptoms with risperidone. Pediatrics. (2010) 125:e1241–5. doi: 10.1542/peds.2009-1815 20385646

[37] Fleming PS Flood TR . Steroid-induced psychosis complicating orthognathic surgery: a case report. Br Dent J. (2005) 199:647–8. doi: 10.1038/sj.bdj.4812929 16311560

[38] Demir T Karacetin G Dogangun B Kocabasoglu N . Report of a case of steroid-induced psychosis and inappropriate sexual behaviour in an adolescent. Pharmacopsychiatry. (2012) 45:77–9. doi: 10.1055/s-0031-1291234 22086740

